# Comparative Evaluation of the Crestal Bone Level Around Pre- and Post-loaded Immediate Endoosseous Implants Using Cone-Beam Computed Tomography: A Clinico-Radiographic Study

**DOI:** 10.7759/cureus.34674

**Published:** 2023-02-06

**Authors:** Janak Wakankar, Sachin B Mangalekar, Pallavi Kamble, Nitin Gorwade, Shashank Vijapure, Priyanka Vhanmane

**Affiliations:** 1 Department of Periodontology, Bharati Vidyapeeth (Deemed to be University) Dental College and Hospital, Sangli, IND; 2 Department of Periodontology, Bharati Vidyapeeth (Deemed to be University) Dental College and Hospital, Sangli , IND

**Keywords:** extraction sockets, alveolar ridges, cone beam computed tomography, endoosseous implant, crestal bone level

## Abstract

Introduction

Dental implants replace missing teeth. Dental implants are surgically placed tooth root replacements that secure prosthetic teeth and bridges. Branemark's original dental implant technique included a mesiobuccal flap and a two-stage approach, needing 6-8 months of recovery following extraction, sterile conditions, machined titanium implants, 3-6 months without stress for osseointegration, and a detachable temporary prosthesis. The restoration would usually be ready a year following the implant surgery. Implant treatment seeks the best function, aesthetics, and complication risk. Implant therapy with low patient morbidity and fast extraction-to-restoration times is a secondary target. Instantaneous implant insertion has made implant dentistry more convenient for patients and clinicians. This study measures bone height before, after, and one month after implant placement using cone-beam computed tomography (CBCT).

Materials and Methods

Participants were selected from oral evaluation candidates. This investigation included 11 people missing front maxillary or mandibular teeth or root components. Diagnostic castings determined the interarch connection before surgery. Alginate maxillary and mandibular arch imprints were cast in Type III dental stone for diagnosis. CBCT scans were taken pre-operatively, post-implant, and post-prosthesis. After the tooth was removed, the empty socket was cleaned up with curettes. An intraoral periapical radiograph and manual probing were done to determine the implant's size. The implant was removed for examination after three months, and healing abutments and gingival formers were placed. Finally, fins were placed. The CBCT images also captured the bone height around the implants. The soft tissue parameters were recorded and evaluated at baseline and one-month following prosthetic loading as plaque index (PI). Radiographic evaluation was done at baseline and one-month following functional loading using CBCT. After one month following functional loading, crestal bone levels were measured again with the help of CBCT using Image J software (National Institutes of Health, Bethesda, Maryland, US).

Results

The sample population had an average age of 42.81 years, with a standard deviation of 13.44 years. Using a paired t-test, we found that the mean PI dropped significantly from pre-loading levels to one-month post-loading levels, with a p-value of less than 0.001. The mean crestal bone level (mesial) evaluated by CBCT at baseline and one-month post-loading was 2.52 ± 1.97 mm and 1.17 ± 1.31 mm, respectively. The mean difference between mean crestal bone loss (distal) at baseline and one-month post-loading was 0.94 ± 1.89 mm, which was not statistically significant. The mean difference between mean crestal bone loss (buccal) at baseline and one-month post-loading was 1.82 ± 1.60 mm, which was statistically significant. The mean difference between mean crestal bone loss (lingual) at baseline and one-month post-loading was 1.91 ± 1.53 which was statistically significant.

Conclusion

CBCT provides all the diagnostic data needed for implant placement; hence, it is recommended.

## Introduction

Modern dentistry strives for healthy dentition to have an optimal function, structural balance, and aesthetic harmony [1]. Patients who lose teeth may experience severe psychological and physical effects. Although periodontitis is primarily responsible for tooth loss, other factors like cavities, trauma, abnormalities during birth, and inherited diseases may also be at play. Dental implants are a good option for those who have had their teeth removed [1]. In 1952, Branemark discovered that osseointegration was feasible using titanium implants. This inspired him to develop and test the first titanium-only dental implant fixtures, which would later come to be known as them [2]. Dental implants are artificial tooth roots that are surgically inserted into patients to act as a stable and long-lasting anchor for a bridge or prosthetic tooth. Zirconia, polymeric materials, or titanium can all be used to make implants. Using a mesiobuccal flap and a two-stage procedure, Branemark's original protocol for dental implant placement called for sterile conditions, machined titanium implants, a healing period of 3-6 months without stress for osseointegration to take place, and a removable temporary prosthesis. Most of the time, it would take at least a year after the implant operation for the restoration to be ready for placement. Due to this, some have questioned whether using a two-stage procedure is actually necessary [2].

The recommended interval between tooth extraction and implant placement is at least six months [3]. The alveolar ridge changes in size during the first three months of recovery after tooth extraction due to a normal physiological process that occurs (such as resorption) [1]. Achieving the best results in terms of function, aesthetics, and complication risk is the main objective of implant treatment. The provision of implant therapy with little patient morbidity and a quick transition from extraction to restoration are the secondary objectives. Historically, only periapical radiographs and panoramic images were used to diagnose implants and develop treatment plans. Computed tomography (CT) and cone-beam computed tomography (CBCT) are now widely recognized as essential for precise implant placement, particularly in the case of complex reconstructions, thanks to advancements in radiography technology. Implant dentistry has been transformed by the invention of the instantaneous implant insertion technique, which is more practical for both the patient and the dentist. Dental implants can now be inserted into fresh extraction sockets, where they are referred to as immediate implants, thanks to improved knowledge of how bone heals around them [4,5].

The alveolar ridges typically recede after tooth loss. The facial side of the ridge exhibits the most bone loss in the horizontal dimension. Another symptom is the loss of vertical ridge height, which is said to be most obvious on the buccal side [6]. Although this time period has not yet been thoroughly studied, the first few months of life are when bone loss is the fastest. The amount of crestal bone loss around endosseous implants placed in fresh extraction sockets and at the one-month post-loading stage is less known despite the critical importance of bone level. In this study, the height of the bone will be measured using CBCT before, after, and one month after an implant is inserted.

## Materials and methods

At the Bharati Vidyapeeth (deemed-to-be-university) Dental College's outpatient clinic in Sangli, patients seeking treatment for missing or loose teeth or root fragments were examined for this study under the IRB number BV(DU)MC&H/sangli/IEC/2019-20/D-19.

Over a period of five months, an x-ray and a clinical investigation were performed on a regular basis. The individuals chosen for participation were those scheduled for a standard oral evaluation. For this analysis, 11 participants who had missing front teeth, either missing root parts, or missing maxillary or mandibular teeth were called back. Inclusion criteria include patients who must be 18 years of age or older. Anterior teeth on the mandible and maxilla should be extracted. Patients who require tooth extractions due to conditions like caries, trauma that does not affect the alveolar bone, root stumps, and teeth with fractured roots and patients with stable periodontal and dental health and good oral hygiene signed informed consent and followed the suggested plaque control and follow-up schedules. Exclusion criteria include patients with systemic conditions that preclude implant placement, those who smoke or chew tobacco regularly, have untreated periodontal diseases, teeth with bony defects, and teeth with significant periapical pathology, the inability to achieve primary implant stability in multirooted teeth, and patients who are unwilling to undergo surgical procedures.

Eleven participants were provided for this study by the Outpatient Department (OPD) at the Bharti Vidyapeeth Dental College. The procedure was carried out by the guide, and the guide was assisted by taking notes and as needed. After receiving information on how the trial would be conducted, the participants gave their informed consent. Each patient's complete medical and dental history was recorded, and any absolute or relative contraindications were noted so that we could learn more about each patient.

Diagnostic casts were prepared prior to surgery to identify the interarch connection. Alginate impressions of the maxillary and mandibular arches were made, and then cast pouring was done in Type III dental stone for diagnosis. CBCT scans with functional loading were done prior to surgery, after implants, and after prostheses.

Strict adherence to asepsis procedures and sterilization protocols ensured the patient's safety throughout the procedure. Using a 25-gauge needle and a five-ml syringe, a local anesthetic was injected into the teeth before the patient was told to rinse with Betadine mouthwash. A crevicular incision was made around the tooth's buccal and palatal surfaces to make the extraction easier. Additionally, the incision extended past at least two adjacent teeth to the crevicular. Beginning at the line angles of the adjacent teeth and continuing all the way to the mucogingival junction, we made vertical incisions. A full-thickness mucoperiosteal flap was reflected using the periosteal elevator. The tooth extraction process was painless. Curettes were used to clean the tooth's socket after it had been extracted. In addition to an intraoral periapical radiograph (IOPA), manual probing was done. The dimensions of the extracted tooth and the distance between the root tips of the teeth next to it were used to determine the size of the implant.

A root-form, threaded collar, two-piece, endosseous, internal hex implant was surgically inserted using the standard surgical technique in either the front maxilla or mandible. Throughout the procedure, all asepsis and sterilization protocols were strictly followed. Osteotomies were carried out repeatedly until the implant site's length and width were ideal. The implant was inserted into the site in accordance with the manufacturer's instructions using an Adin torque wrench. The primary stability of the implants was assessed following insertion, and the implant mount and cover screw were subsequently installed. Once the implants were put in, the flaps were later sewn back together. The surgical site was cleaned using saline irrigation. Additionally, the patient received medication and post-operative care instructions. Following that, patients were referred for a CBCT scan to measure their crestal bone height. Seven days after surgery, patients were contacted again to have their stitches removed and to get a physical examination.

Both oral and written postoperative instructions were given to every patient. Amoxicillin and clavulanic acid were given to all patients twice daily for five days. For five days, 325 mg of paracetamol and 400 mg of ibuprofen were given to relieve pain. Patients were instructed to apply a 0.2% chlorhexidine solution once daily for two weeks. If recovery had gone well, patients were called back in after seven days to have the sutures taken out. The implant was removed for inspection after three months, and gingival formers and healing abutments were placed. The last prostheses were affixed in functional occlusion after four months. One month after prosthesis loading, the clinical examination was repeated, and the plaque index (PI) was measured. The bone height surrounding the implants was also captured by the CBCT images. The following soft tissue parameters were measured and assessed as the PI at baseline and one month after prosthetic loading: Turesky-Gilmore-Glickman modification of the Quigley-Hein plaque index.

Utilizing CBCT, radiographic evaluation was performed at baseline and one month after functional loading. Using the first thread of the implant as a standard reference point, the circumference of the crestal bone level around the implant was measured in all directions (mesial, distal, buccal, and lingual). Crestal bone levels were once more measured using CBCT and Image J software (National Institutes of Health, Bethesda, Maryland, US) a month after functional loading.

IBM SPSS Statistics for Windows, Version 20 (Released 2011; IBM Corp., Armonk, New York, United States). was used to calculate the statistics. When the data were normal, the independent t-test, also referred to as the paired t-test, was used to compare means or values within the same group or sample. When the p-value was less than 0.05 (p0.05), we considered the result to be statistically significant, and when the p-value was less than 0.05, we created a 95% confidence interval.

## Results

Demographic details of the patients are explained in Table 1.

**Table 1:** Demographic Details of the Patients CBCT: Cone-beam computed tomography

Patients	Age	Gender	Plaque Index	Plaque Index	CBCT Readings at the Time of Implant Placement				CBCT Readings One Month Post-prosthesis			
			Baseline	One Month Post-loading	Mesial	Distal	Buccal	Lingual/Palatal	Mesial	Distal	Buccal	Lingual/Palatal
1	43	Male	2	1.2	2.21	2	-4.45	3.71	2.64	2.48	-3.98	2.62
2	43	Male	2	1.2	-2.34	1.96	2.79	1.02	2.26	1.75	0.38	1.16
3	45	Female	2	1.5	5.54	2.32	4.08	2.99	5.47	-3.39	-1.41	-0.83
4	55	Male	2	1	2.14	2.02	3.8	2.74	1.25	1.57	1.64	1.88
5	55	Male	2	1	2.46	2.24	3	2.29	0.42	0.71	1.15	1.1
6	58	Male	2	1	2.11	2.21	4.25	5.32	1.38	2.53	1.98	0.65
7	58	Male	2	1	2.22	2.19	4.21	5	1.66	4.01	1.22	1.16
8	21	Female	2	1.2	2.82	2.74	1.53	2.97	1.32	1.4	1.22	0.94
9	21	Female	2	1.2	2.67	2.68	2.12	2.49	2.04	1.84	1.78	1.56
10	36	Female	2	1.3	4.82	4.59	0.88	4.4	2.94	2.78	-0.42	3.48
11	36	Female	2	1.3	3.15	2.36	1.74	2.47	1.76	1.2	0.34	0.57

The test population age distribution is shown in Table 2.

**Table 2:** Age Distribution of the Study Population SD: Standard deviation; SE: standard error

	Mean	SD	SE	Minimum	Maximum
Age (in years)	42.81	13.44	4.05	21.0	58.0

There was a bare minimum of 21, and a hefty maximum of 58, for participation. Researchers found that the sample population had an average age of 42.81 years, with a standard deviation of 13.44 years.

We can see how evenly men and women make up the sample size. Eleven participants filled out the research. Six of the eleven participants were male (54.5%) and five were female (45.5%). Patients of either sex were included in the analysis without a difference (Table 3).

**Table 3:** Gender Distribution of the Study Population

	Frequency (n)	Percentage (%)
Male	6	54.5%
Female	5	45.5%

The average PI before prosthesis loading and after one month is shown in Table 4.

**Table 4:** Mean Plaque Index at Baseline to One Month Post-loading SD: Standard deviation; SE: standard error

Variable	Baseline Mean	One Month Mean	Mean Difference ± SE		P Value
Mean Plaque Index	2.0	1.17	0.82 ± 0.16		0.001**

With a mean difference of 0.82 between the PI scores of the study population before and after loading, the average score was 2. Using a paired t-test, we found that the mean PI dropped considerably from pre-loading levels to one-month post-loading levels, with a p-value of less than 0.001.

The comparison of crestal bone levels at baseline and one-month post-loading using CBCT is shown in Table 5.

**Table 5:** Comparison of Crestal Bone Levels at Baseline and One Month Post-loading Using CBCT SD: Standard deviation; SE: standard error; CBCT: cone-beam computed tomography

Crestal Bone Level	Baseline Mean	One Month Mean	Mean Difference ± SE	P Value
Mesial	2.52	1.17	0.42 ± 0.55	0.459
Distal	2.48	1.53	0.94 ± 1.89	0.129
Buccal	2.17	0.35	1.82 ± 1.6	0.004
Lingual	3.2	1.29	1.91 ± 1.53	0.002

The mean crestal bone level (mesial) evaluated by CBCT at baseline and one-month post-loading was 2.52 ± 1.97 mm and 1.17 ± 1.31 mm, respectively. The mean difference between crestal bone loss (mesial) at baseline and one month post-loading was 0.42 ± 0.55 mm, which was not statistically significant when measured by the paired ‘t-test with a value of p = 0.459. At baseline and one month after loading, CBCT measurements of the distal crest of the skeleton showed values of 2.48 mm and 1.53 mm, respectively. The mean difference between mean crestal bone loss (distal) at baseline and one-month post loading was 0.94 ± 1.89 mm, which was not statistically significant when measured by the paired ‘t-test with a value of p = 0.129. The mean crestal bone level (buccal) evaluated by CBCT at baseline and one month post-loading was 2.17 ± 2.48 mm and 0.35 ± 1.75 mm, respectively. The mean difference between mean crestal bone loss at baseline and one month post loading was 1.82 ± 1.60 mm, which was statistically significant when measured by the paired ‘t-test with a value of p = 0.004. The mean crestal bone level (lingual) evaluated by CBCT at baseline and one month post loading was 3.20 ± 1.27 and 1.29 ± 1.12, respectively. The mean difference between mean crestal bone loss at baseline and one month post-loading was 1.91 ± 1.53 which was statistically significant when measured by the paired ‘t’ test with a value of p=0.002.

## Discussion

The root cementum, periodontal ligament, and bundle bone support the tooth and together they make up a single, functional structure. The biting forces may be more evenly distributed across the jaw when the crown transmits them to the alveolar bone. Both single-tooth extractions and multiple-tooth extractions result in a series of adaptive alterations in the nearby soft and hard tissues, which eventually cause the edentulous site to recede. Compared to the lingual and palatal sides of the ridge, this appears to occur more frequently on the buccal side. Branemark's original dental implant procedure required sterile conditions, a mesiobuccal flap, and a two-stage approach, all of which took place six to eight months after extraction [7]. A detachable interim prosthesis will be needed for a significant period of time because osseointegration needs a stress-free healing period of three to six months. From the time of implant surgery, until the last restoration was put in, it would take at least a year. The Branemark protocol, which employs a two-stage technique, raises additional problems like volume loss of the alveolar bone, protracted edentulism, a higher volume of surgical procedures, and psychological effects on the patient [7,8]. The first year after a tooth extraction is when this change in the bone along the alveolar ridge is most obvious [9].

Immediate implant placement, in which a dental implant is inserted into the extraction socket at the time of extraction, is a method of tooth replacement that is growing in popularity [10]. The panoramic, lateral cephalometric, and intra-oral periapical extraoral radiographs make up the standard projection. Advanced radiography techniques include CT, spiral CT, CBCT, and interactive computer-guided implantology [11,12]. Because it provides a precise measurement of the distance between the alveolar ridge and important structures like the mandibular canal, the maxillary sinus, etc., CBCT is helpful in defining the safety zone. Measurements of the buccal and lingual bone plates were demonstrated by Schulze et al. using CBCT [13].

According to research, the average bone loss around implants is 1 mm in the first year following prosthesis placement and then stays constant at 0.1 mm each year after that. Premature bone loss around the implant can result from a number of factors, including surgical stress from overheating, implant location, the development of biological space, surgical technique, and implant design. Due to the formation of devitalized bone around the implant as a result of the osteotomy procedure used to place osseointegrated implants, early peri-implant bone loss has been linked to this procedure. Because blood flow is interrupted and heat is produced during osteotomy, a dead spot develops in the cortical bone [14].

In the current investigation, plaque accumulation on the front side of teeth next to dental implants was also examined. Similar to the current findings, Koh et al. reported that after four months, all patients still had low levels of plaque accumulation [15]. Each participant in a study by Behneke et al. that monitored PI around implants for three years at intervals of six months reported that values remained within physiologic ranges during the entire observation period, indicating that healthy peri-implant conditions predominated [16].

In the current study, CBCT was used to measure the crestal bone levels around immediate implants. In cases of bone loss, radiographs are a useful addition to clinical examinations. In order to evaluate the bone density of peri-implantitis patients, radiographs are frequently used. Despite some drawbacks, 3D imaging techniques like CT scans may do away with the drawbacks of 2D radiography. The rate of crestal bone loss in the region can be used to determine the long-term success of a dental implant. A crucial success factor was suggested as crestal bone loss of less than 1.5 mm in the first year following loading and less than 0.2 mm annually after that [17].

As determined by CBCT evaluation of marginal bone levels and horizontal ridge diameters, Cardaropoli et al. [18] and Vera et al. [19] discovered that marginal bone levels were constant after one year of implant placement in new extraction sockets. The flat breadth surrounding the top was largely unaltered. Another systematic review of buccal bone changes around single embeds placed immediately (10 days), 90 days later, and 1.5 years after tooth extraction was carried out by Schropp et al. using CBCT [20]. They discovered that the mean buccal bone loss after 10 years was 2.39 mm for implants that were placed right away, 2.22 mm for implants that were placed later, and 1.85 mm for implants that were loaded later [20]. In the current study, dental implants were placed shortly after teeth were extracted. The effects of tooth extraction on the surrounding hard and soft tissues' dimensions have been studied. To gauge how much the extraction sockets have changed as they recover, several techniques have been used. Over the first four months after healing, the buccal-lingual ridge shrinks by about 5-7 mm [21], with a loss of 2-4.5 mm of vertical bone height. It is possible to reduce the number of surgical procedures performed, the length of time required for treatment, the preservation of alveolar bone, the preservation of ideal soft tissue contours, the improvement of implant placement, the simplification of prosthetic design, and the attitude of patients toward dental care.

In the study by Raes et al., after one year, the average circumferential bone level surrounding implants implanted in a new extraction socket with immediate loading was 0.21 mm, according to a CBCT evaluation. They concluded that CBCT is more precise than IOPA [22]. Using standardized digital periapical radiographs. The study by Tadi et al. on crestal bone loss in immediate implant implantation found an average loss of 0.80 mm at one month, 1.03 mm at three months, and 1.23 mm at six months [23]. Huber et al. carried out additional investigation into how implants placed in fresh extraction sockets affected the crestal bone level, finding a mean loss of 0.49 mm after a year [24].

The current study discovered a statistically significant reduction in the thickness of the buccal and lingual bones. These results are in line with those of the study by Sanz et al. which used tapered or cylindrical implants and measured the horizontal and vertical ridge dimensions 16 weeks after implant implantation into an extraction socket and discovered a significant loss of buccal/palatal bone [25]. In our study, bone loss was quantified and found to be insignificant in the distal and mesial regions. When Schropp et al. compared the bone healing and crestal bone changes after immediate and delayed placement of implants, they discovered similar results, while the height of the buccal and lingual bone crests at the extraction site was decreased, the mesial and distal socket walls remained largely unaltered [26]. The mesial and distal portions of the socket changed less than the buccal and lingual portions after a single tooth was pulled, and an implant was immediately placed.

Therefore, it is possible to say that CBCT may be crucial in determining how much bone is around an implant after surgery within the context of the current study. The CBCT has some disadvantages, including a high price, more radiation exposure than IOPA, and restricted accessibility. However, given the numerous benefits, this is frequently overlooked. To the best of our knowledge, this is the first study to evaluate crestal bone levels near immediate implants using CBCT technology. To further support our study's findings, larger clinical studies with longer follow-up times should be conducted.

The study has some limitations of its own. To come to a final conclusion, more extensive clinical trials comparing the crestal bone levels around immediate and delayed implant insertion with a longer follow-up period are required, even though this research has produced some encouraging preliminary results.

## Conclusions

Preoperative planning and meticulous monitoring of the healing process are needed to assess osseointegration. CBCT provides all the diagnostic data needed for successful implant placement. CBCT analysis was performed at baseline and one month post-loading. The mean crestal bone mesial and distal showed a non-significant difference while mean crestal bone buccal and lingual had a significant difference. So, inserting implants immediately helps anticipate treatment outcomes.
